# Informatics-Driven Design of Superhard B–C–O Compounds

**DOI:** 10.1021/acsami.3c18105

**Published:** 2024-02-17

**Authors:** Madhubanti Mukherjee, Harikrishna Sahu, Mark D. Losego, Will R. Gutekunst, Rampi Ramprasad

**Affiliations:** † School of Materials Science and Engineering, Georgia Institute of Technology, Atlanta, Georgia 30332, United States; ‡ School of Chemistry and Biochemistry, Georgia Institute of Technology, Atlanta, Georgia 30332, United States

**Keywords:** superhard, Vicker’s hardness, B−C−O chemical space, machine learning (ML), DFT, crystal structure search, elastic moduli

## Abstract

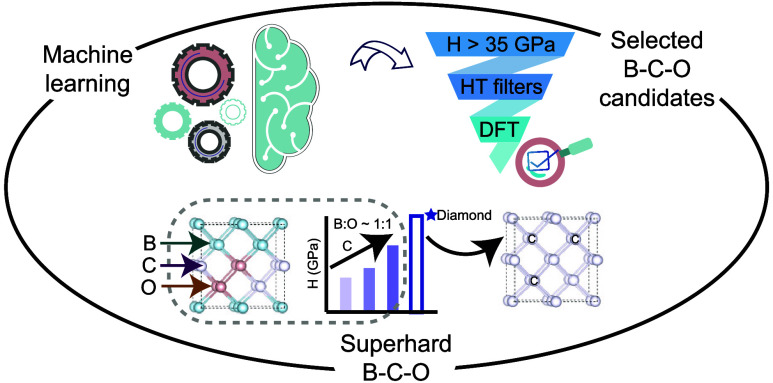

Materials containing B, C, and O, due to the advantages of forming strong covalent bonds, may lead to materials that are superhard, i.e., those with a Vicker’s hardness larger than 40 GPa. However, the exploration of this vast chemical, compositional, and configurational space is nontrivial. Here, we leverage a combination of machine learning (ML) and first-principles calculations to enable and accelerate such a targeted search. The ML models first screen for potentially superhard B–C–O compositions from a large hypothetical B–C–O candidate space. Atomic-level structure search using density functional theory (DFT) within those identified compositions, followed by further detailed analyses, unravels on four potentially superhard B–C–O phases exhibiting thermodynamic, mechanical, and dynamic stability.

## 1 Introduction

A wide variety of practical applications require superhard materials,^1−4^ defined as materials with a Vicker’s hardness value exceeding 40 GPa. For reference, the hardness of diamond is 93 GPa, and that of Aluminum is 0.167 GPa. Hardness (H) is measured by the amount of surface deformation upon indentation originally developed by Smith and Sandland in 1921.^5^ It is typically performed by a specially designed tip that indents the material to evaluate its ability to resist deformation.^6,7^ These experiments are intricate and expensive to perform. Although diamond is the hardest known material, it has several limitations, such as instability to oxidation above 800 °C, and reactivity towards iron-containing compounds.^1,8^ These drawbacks have fueled ongoing efforts to find newer superhard materials with improved thermal and chemical stability.

Compounds containing light elements, such as B, C, N, and O are some of the most promising candidates in this regard. These elements can form strong covalent bonds, which are critical for superhard materials. Several B–C–O phases and BC_*x*_, and BC_*x*_N materials with Vicker’s hardness greater than 30 GPa have been synthesized successfully at high pressure and temperature.^9−14^ In particular, recently synthesized ternary B_1_C_2_N_1_ with a hardness of 76 GPa is the second hardest known material.^15−18^ Similar to B–C, C–N, C–C, and B–N covalent bonds in superhard B–C–N compounds, strong B–O and C–C covalent bonds can also form in B–C–O compounds.^12,14,19^ Indeed, several B–C–O compounds have been proposed using density functional theory (DFT) based computational approaches to display high hardness, including B_2_C_*x*_O (*x* = 1, 2, 3, 5), B_4_CO_4_, two-dimensional (2D) B–C–O alloys, and B_*x*_O. Nevertheless, exhaustive searches of the B–C–O space and ascertaining that these compounds display thermodynamic, mechanical, and dynamical stability are nontrivial using purely DFT methods.

Data-driven machine learning (ML) approaches have proven to be exceptionally efficient in exploring large chemical and configurational spaces of materials. ML and/or DFT approaches have been extremely successful in identifying inorganic and organic materials with various physical and chemical properties, such as band gaps, conductivities, good catalytic performances, high/low thermal conductivity materials, and many more.^20−42^ ML models have also been developed to predict hardness by utilizing structural information such as volume, crystal symmetry, elemental information such as melting point and other input such as cohesive energy.^43−46^ Obtaining these inputs for a new search space requires additional prior knowledge. These drawbacks were overcome by Chen et al. by using features derived purely from the chemical composition to predict the hardness, which has led to the discovery of a few superhard B–C–N compounds.^47^ Their study used the elastic moduli, such as the bulk modulus (*K*) and shear modulus (*G*), as proxy properties to overcome the difficulty of obtaining hardness directly from DFT. Although these models do not capture the influential factors such as load dependency on the hardness, thus offering an opportunity for improvement.^48^ The utilization of elastic moduli as surrogate properties has demonstrated promising potential as an alternative approach to address the challenges associated with predicting hardness.

The elastic moduli are correlated to Vicker’s hardness^45,46^ and can reliably be determined through first-principles calculations. The linear correlation between elastic moduli and hardness was well-established by several empirical models that serve as alternatives to challenging estimations of hardness. Such empirical models include Teter’s model, Chen’s model, and Tian’s model.^49−51^ Teter’s model, based on the observed correlation between shear modulus and hardness, offers valuable insights; however, it lacks the consideration of plastic deformation, crucial for understanding hardness. Tian’s model incorporates considerations of plastic deformation through Pugh’s ratio (*G*/*K*). Pugh’s ratio includes both bulk (*K*) and shear (*G*) moduli, which are the measure of resistance to uniform compression and shear deformation, respectively. This inclusion is particularly important, as it provides a more accurate representation of material behavior under stress. While Teter’s and Chen’s models yield physically inaccurate hardness, particularly for ductile materials, Tian’s model has shown reasonable accuracy in determining hardness for ductile and brittle materials. Tian’s model is a revised formula based on microscopic evaluation of hardness. In addition, the hardness obtained from Tian’s model exhibits good agreement with experimental values.^50−56^ The mathematical form of the model is
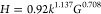
1where *k* is known as Pugh’s ratio (*G*/*K*), representing plastic deformation.

**Figure 1:**
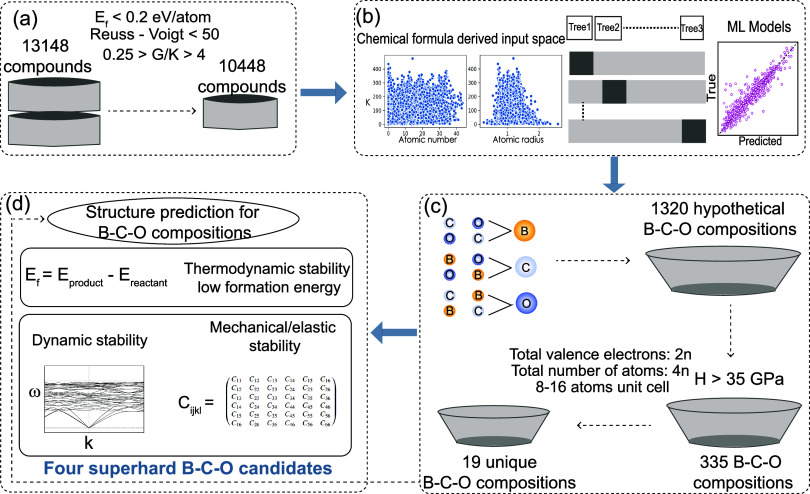
Schematic workflow for the data-driven discovery of superhard B–C–O compounds: (a) Data acquisition of 13148 compounds having DFT computed elastic moduli and three applied criteria to select a suitable training set, (b) regression-based ML model using cross-validation and chemical formula derived features on a set of 10448 compounds, (c) employed models on a set of B–C–O composition, leading to 335 B–C–O compositions with hardness more than 35 GPa, and (d) three high-throughput filters enforced superhard B–C–O compositions.

In the present work, we demonstrate a design framework for identifying novel superhard B–C–O compounds, as shown in Figure 1. This framework integrates the development of two machine-learning models to predict bulk and shear moduli of materials given just their compositions, the estimation of hardness by using Tian’s model, and employing a crystal structure prediction algorithm (USPEX) in conjunction with first-principles calculations for structure prediction and validation of the ML identified compositions. In this study, Tian’s model is used for defect-free systems. Although the methodology is not entirely novel, this study is distinctively focused on exploring the B–C–O phases. Existing models were not as effective in this area, prompting us to develop new models that more accurately capture the distinct chemical properties of the B–C–O systems. The initial data set to train the ML models comprises bulk and shear moduli computed using DFT for 13,148 compounds. From this initial pool, 10,448 compounds were selected based on the formation energy and elastic modulus as the guiding criteria, as depicted in Figure 1(a). Subsequently, two ML models were developed using the chemical compositions of these chosen compounds as input features, as illustrated in Figure 1(b). These models were employed on a set of unexplored B_*x*_C_*y*_O_*z*_ compositions, revealing several B–C–O compositions possessing a predicted hardness of more than 40 GPa, qualifying them as potential superhard compositions. A total of 19 unique compositions were identified by applying three filters, as displayed in Figure 1(c). Further, the equilibrium atomic symmetries of these chosen compositions were determined, followed by an exhaustive evaluation of their thermodynamic, dynamic, and mechanical stability, combined with the determination of their elastic properties, as demonstrated in Figure 1(d). Finally, four superhard compositions with a hardness of more than 40 GPa, namely, B_1_C_10_O_1_, B_4_C_8_O_4_, B_2_C_9_O_1_, and B_2_C_8_O_2_ were identified, which are mechanically and dynamically stable and exhibit relatively low formation energy. Furthermore, we implemented SHAP analysis, which provided both global and local insights into the impact of various features on predicted hardness, as detailed in the Supporting Information through various SHAP plots. It is hoped that these superhard B–C–O compositions will go through validation by physical synthesis and characterization.

## 2 Methods

An overview of the steps followed in this work, including data curation, development of ML models, atomic structure determination, and stability assessment, are explained in this section.

### 2.1 Data Set and Features

The properties of interest in this study that are correlated to hardness are bulk and shear moduli. Initially, 13,116 samples were collected from Materials Project containing DFT calculated bulk and shear moduli values in the ranges of 0–550 GPa.^47,57^ Previously developed ML models using these training data by Chen et al. show inaccurate predictions of bulk and shear modulus when applied to not-so-well-explored B–C–O compounds. For instance, the previous models predict bulk and shear moduli of 174.3 and 102.1 GPa, respectively for B_2_C_3_O_1_, which are considerably different from the previously reported DFT calculated values of 322 and 302 GPa, respectively.^58^ Recognizing the lack of relevant ML-training data, we have augmented the data set by adding 32 DFT computed elastic constants for certain poorly predicted B–C–O compositions and those which are available in the previous reports.^13,58,59^ The DFT modulus values demonstrate the Voigt-Reuss-Hill average moduli. In order to manage the accuracy of the models, training data were selected by setting a few criteria. These include samples with formation energy less than 0.2 eV/atom, Voigt–Reuss modulus difference larger than 50 GPa, and *k* (*G*/*K*) to be within 0.25 to 4, as shown in Figure 1(a). These yield a total of 10,448 samples out of a total of 13,148 samples for developing the ML model. The features derived from the chemical composition (Figure 1(b)) are elemental, orbital, and electronic levels information. These include mean, range, and fraction weighted atomic weight, row, and column number in the periodic table, atomic number, atomic radius, electronegativity, and the number of s and p orbital electrons. There are a total of 60 features utilized in training the models.^47^

### 2.2 ML Model

ML models were developed for bulk (*K*) and shear (*G*) moduli using a random forest algorithm. The models were trained on 90% of the total data set with 20 random trials. The training samples were divided into training and validation sets. These models were tested on the remaining 10% of the data. Further, the predictions were made on the final production models and used to estimate the hardness (H) subsequently using eq 1. The training validation set was used for the grid search with 10-fold cross-validation, which resulted in a tree depth of 30 and 40 layers (with 160 estimators) for bulk and shear modulus models, respectively. The performance of these developed models was evaluated by using regression metrics, namely root-mean-square error (rmse) and the coefficient of determination (*R*^2^). The best-performing model was selected based on the highest *R*^2^ and the lowest train/test rmse after optimizing the hyperparameters over all of the random trials.

### 2.3 Crystal Structure Prediction

The compositions with predicted hardness of over 35 GPa were considered further for a structure search. In order to make the structure search robust, the Universal Structure Predictor: Evolutionary Xtallography (USPEX) was used, which has been successful in finding out energetically favorable structures from given compositions.^60,61^ USPEX finds stable and metastable phases of given compositions by using the *ab initio* free energy of the locally optimized structure as the fitness value. The evolutionary optimization creates new structures by using random (20%), mutation (30%), and heredity (50%) operators. In order to focus on discovering superhard compounds, an external pressure of 15 GPa was applied during the structure search. A small but finite pressure helps to avoid low-hardness graphite-like structures.

The optimized structures obtained from the USPEX search were fully relaxed by using DFT without external pressure. DFT calculations were performed using the projector augmented wave (PAW) method and generalized gradient approximation (GGA) functional in Perdew–Burke–Ernzerhof (PBE) formalism as implemented in VASP.^62,63^ The kinetic energy cutoff was kept at 520 eV, and the kpoints were sampled by a centered Monkhorst–Pack mesh with a resolution of 0.04. The convergence criteria for structure relaxation and self-consistency calculations were set to 10^–5^ eV.

### 2.4 Stability Assessment for Predicted Structures

For the fully relaxed structures, the strain–stress method was utilized as implemented in VASP to obtain the elastic tensor. More stringent energy criteria of 1 × 10^–8^ eV were used to ensure a well-converged elastic tensor. The eigenvalues of the elastic tensor and the Voigt-Reuss-Hill (VRH) averaged bulk (*K*), and shear (*G*) were computed via the MechElastic library.^64^ The dynamic stability of the structures was analyzed by calculating the minimum eigenvalue of the dynamical matrix obtained from the Phonopy package.^65^ The required interatomic force constants were calculated using a supercell size of 1 × 4 × 2. To obtain well-converged phonon dispersion, a k-grid of 6 × 6 × 7 and an energy cutoff of 500 eV with a strict energy convergence criterion of 10^–6^ eV were used. The long-range electrostatic interactions were considered by calculating the Born effective charges, as implemented in density functional perturbation theory.

## 3 Results

### 3.1 Identifying Superhard B–C–O Compositions

**Figure 2:**
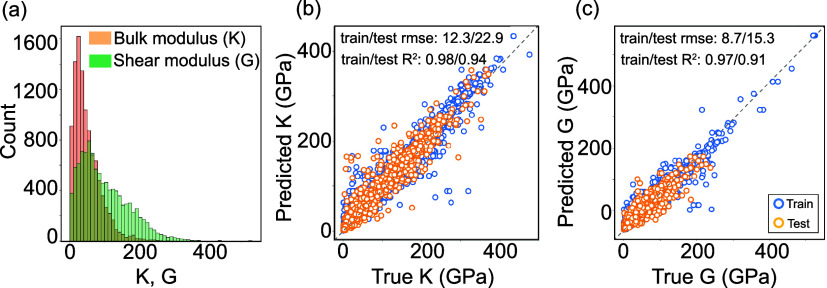
Distribution of the samples and performance evaluation of random-forest-based training models: (a) Histogram of bulk (*K*) and shear (*G*) moduli for 10,448 samples, scatter plots of (b) bulk and (c) shear moduli predictions using chemical composition derived features.

**Figure 3:**
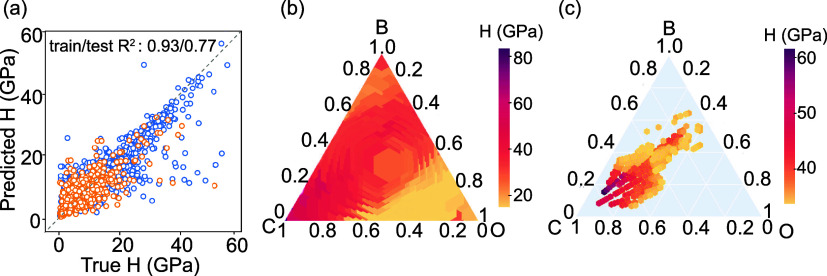
ML predicted hardness for the hypothetical B–C–O candidate space: (a) Scatter plot showing the predicted and true hardness as obtained from Tian’s formula with *K* and *G* as inputs, (b) B–C–O ternary graphs for hardness (H) as estimated from predicted *K* and *G*, and (c) an organized ternary graph indicating a 1:1 B–O ratio can lead to several superhard compositions, for instance, B_1_C_10_O_1_ (*H*_pred_ = 52.71 GPa).

The hardness of any material is closely correlated to the elastic properties, such as the bulk and shear moduli of the system. Therefore, this study considers ML predictions of elastic moduli as proxy properties, which were further used to estimate the hardness by utilizing Tian’s model. The histograms of DFT computed bulk (*K*_DFT_) and shear (*G*_DFT_) moduli are shown in Figure 2(a) for 10,448 samples. These samples are thermodynamically favorable (i.e., formation energy <0.2 eV), and they possess *k* (*G*/*K*) between 0.25 and 4.0. *k* > 0.25 ensures the resulting hardness will not be very small. Structures with *k* > 4 indicate exceptional hardness, surpassing 200 GPa, which necessitates high-pressure conditions for their accurate computation and, therefore, have been excluded from this study. Next, two prediction models were developed for bulk and shear modulus, for which the model parameters were tuned during the learning process. Optimized hyperparameter values for the developed RF models are listed in Table S1. The optimized training model for the bulk modulus results in the lowest train/test rmse of 12.3/22.9 GPa with *R*^2^ 0.98/0.94, as shown in Figure 2(b). The second training model for predicting shear modulus also shows reasonable performance with the lowest train/test rmse of 8.7/15.3 with *R*^2^ 0.97/0.91, as shown in Figure 2(c). The estimation of the hardness was done by using the ML-predicted elastic moduli, for which the performance is shown in Figure 3(a). Relatively poor accuracy with *R*^2^ of 0.77 can be attributed to the fact that the prediction of hardness was not directly obtained but rather determined from the empirical formula, as shown in eq 1. We note that developing a single prediction model for hardness can be limited by available experimental data, and using an empirical model directly can affect the accuracy of the model.

After obtaining the optimized prediction models, we next employ these models on a set of B–C–O compositions, which were generated by enumerating a series of B_*x*_C_*y*_O_*z*_ with *x*, *y*, *z* ∈ 1,2,···9. In Figure 3(b), the ternary plot showcases the predicted hardness for the B–C–O compositions, where the vertices correspond to elemental compounds. Notably, the pure carbon phase exhibits a predicted hardness of 93 GPa, indicating its resemblance to the cubic diamond phase. The corresponding zoomed-in portion of Figure 3(c) focuses on compositions with a 1:1 B:O ratio and higher carbon content, wherein hardness values exceeding 40 GPa are observed. For instance, the predicted hardness values for compounds B_2_C_3_O_1_ and B_2_C_5_O_1_ are 36.1 and 55.5 GPa, respectively. These predictions align well with findings reported in previous studies.^58^ The targeted search resulted in the identification of 335 compositions with hardness values exceeding 35 GPa. The plot also reveals high hardness predictions near the pure oxygen phase. This phenomenon can be attributed to the characteristic behavior of oxygen-dominated compounds, which often exhibit a small bulk modulus, resulting in an amplified *k* = *G*/*K*. As a consequence, these predictions may give a misleading impression of higher hardness stemming from an apparent overestimation rather than an accurate representation.

### 3.2 Atomic Structure Prediction and DFT Validation

Next, an atomic-level structure search was performed by using USPEX. The structure search was primarily focused on the fixed compositions around the B:O ratio ∼1:1, characterized by the predicted hardness exceeding 35 GPa, leading to a total of 335 compositions.

To address computational constraints, three high throughput filters were employed to narrow the selection to the most promising compositions. First, compositions with an even number of total valence electrons were screened, ensuring the stability and insulating nature of the resulting phases. The rationale behind prioritizing an insulating phase primarily stems from the bonding characteristics. Short and strong covalent bonding, where electrons are tightly bound, inherently leads to insulating behavior due to the restricted mobility of electrons, unlike in conductive materials. Simultaneously, such bonding results in higher hardness by ensuring a rigid and stable crystal structure. Second, to prioritize structures resembling diamond, the total number of atoms within the compositions was restricted to 4*n* (*n* = 2, 3, and 4). Additionally, the maximum number of atoms considered for the structure search was limited to 8, 12, and 16 atoms per unit cell. Additionally, to avoid redundancy within the selected compositions, only one representative structure was chosen from similar compositions, such as those with identical ratios (e.g., B_2_C_4_O_2_ and B_4_C_8_O_4_). These employed filters led to the identification of 19 unique B–C–O compositions having number of atoms 12 and 16, meeting the desired criteria and exhibiting promising characteristics for further analysis and exploration. The down-selection process is demonstrated in Figure S1. Based on the insights provided by the ML model, which indicated that a higher carbon content in the compositions results in increased hardness, two compositions were excluded from the initial set of 19. These two compositions had lower carbon content compared to the elements B or O. Two of the compositions within the data set had been previously studied^58^ and were included in the training set of the ML model. Consequently, these compositions were excluded from the analysis to ensure an independent validation. As mentioned previously, the ML predictions aligned with the hardness of these two compositions, further validating the accuracy and reliability of the ML model. By excluding these compositions, the focus was narrowed to compositions that align more closely with the desired carbon content for enhanced hardness. Among the remaining 15 B–C–O compositions (listed in Table S2), a structure search was conducted to explore, identify, and validate the most promising crystal structures within these compositions. The structure selection criteria are summarized in SI.

**Figure 4:**
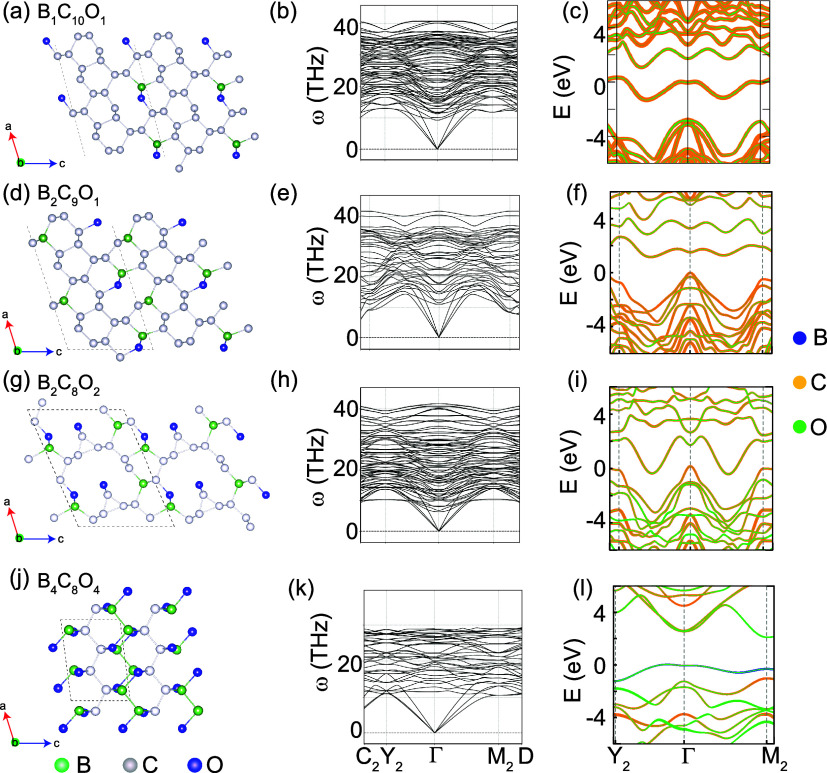
Evolutionary algorithm and first-principles calculations based structure search, stability assessment, and electronic structure properties for identified superhard compositions: (a, d, g, j) crystal structures (left panels), (b, e, h, k) phonon dispersions (middle panels), and (c, f, i, l) electronic band structures (right panels) for ML predicted compositions i.e B_1_C_10_O_1_, B_4_C_8_O_4_, B_2_C_9_O_1_, and B_2_C_8_O_2_, respectively. The structures are represented along the *b*-axis (with the *b*-axis perpendicular to the page).

The USPEX structure search was carried out by applying a small and finite pressure of 15 GPa. This facilitated the identification of more stable structures with reduced volumes, thereby favoring phases with higher hardness. We start the structure search for fixed compositions consisting of 12 atoms. Leveraging the prior knowledge of B_1_C_10_N_1_ compositions exhibiting high hardness, as established by Chen et al.,^47^ we explored the potential candidacy of B_1_C_10_O_1_. Although it does not meet the criteria set by the high-throughput (HT) filters, we conducted a structure search for B_1_C_10_O_1_ utilizing a single-formula unit cell, motivated by its potential relevance and significance in the context of our study. The structure search demonstrated an energetically metastable nonlayered B_1_C_10_O_1_, which crystallizes in a monoclinic phase, as shown in Figure 4(a). The DFT-calculated hardness of 55.5 GPa agrees well with the ML-predicted hardness of 52 GPa.

To explore the ML-informed 12-atom compositions (Table S2), we utilized the stable reference structure of B_1_C_10_O_1_ and derived six compositions by replacing carbon with additional boron and oxygen. After conducting symmetrization and geometric optimization, only three structures, namely, B_2_C_9_O_1_, B_2_C_8_O_2_, and B_4_C_7_O_1_ were successfully obtained. Despite conducting additional structure searches using USPEX, we did not discover any structures with lower enthalpy compared to the identified ones. All three structures exhibit sp^3^ bonding and crystallize in the monoclinic phase, as depicted in Figures 4(d,g), and S2(a). The ML-predicted hardness for these compounds was further validated by calculating the hardness using DFT. The calculated and ML-predicted bulk and shear moduli and estimated hardness for these compounds are summarized in Table S3. B_2_C_9_O_1_ and B_2_C_8_O_2_ are classified as superhard compounds with the calculated hardness of 57.4 and 40.4 GPa, respectively, while B_4_C_7_O_1_ has a relatively lower calculated hardness of 30.9 GPa.

Next, we redirected our investigation towards compositions composed of 16 atoms. Motivated by the exceptional hardness of the second hardest known material, B_1_C_2_N_1_, we embarked on an exploration of compositions within the B–C–O system that exhibit a similar ratio. Thus, leveraging the promising compositions identified through our ML model, we first considered the search for B_4_C_8_O_4_ with 16 atoms in the unit cell. In a recent study, a superhard B_1_C_2_O_1_ material was investigated, derived from a novel three-dimensional (3D) carbon allotrope *m*-C_8_.^66^ Building upon this discovery, we constructed a B_4_C_8_O_4_ structure from a 1 × 1 × 2 supercell of diamond and introduced substitutions by replacing eight carbon atoms with four boron and four oxygen atoms. Among the structures generated through USPEX and atomic substitution, we observed that the one with atomic substitution exhibited a lower enthalpy compared with the structures generated solely through USPEX. This prompted us to prioritize the structure with atomic substitutions for further analysis and exploration. The optimized structure (Figure 4(j)) revealed sp^3^ bonding within the monoclinic phase. After DFT calculations were performed for elastic constants, a hardness of 45.9 GPa was obtained, closely aligned with the ML-predicted hardness value of 41.19 GPa. These promising results indicate the potential of this superhard material for various applications and warrant further exploration and investigation. Upon employing the rule of atomic substitution, we generated structures corresponding to eight compositions consisting of 16 atoms. However, our search within the explored space did not yield any other stable configurations.

### 3.3 Determining the Stability of the Identified Phases

For practical applications, assessing thermodynamic, dynamic, and mechanical stabilities is crucial. To evaluate these properties, the formation energy, phonon spectra, and elastic constants are calculated for these compounds. The formation energies for these compounds range from 0.11 to −0.49 eV/atom as listed in Table S3, which are comparable or relatively less than previously studied B–C–N and B–N–O superhard compounds.^47,67^ The absence of imaginary frequencies in phonon dispersions, as shown in Figures 4(b,e,h,k) and S2(b), implies these structures as dynamically stable. The electronic band structures with atomic contributions for the four superhard compositions are shown in Figure 4(c,f,i,l). All these compounds are mechanically stable, which was verified by Born’s criteria, as explained in SI. These systems are novel and show potential. While these four compositions demonstrating good stability are emphasized, all 15 B–C–O compositions listed in SI provide a resource offering potential candidates for further exploration. Recent experimental pathways for synthesizing superhard B–C–N and other B–C–O phases open up the possibility for successfully realizing such B–C–O compounds.^17,59,68−70^

The findings presented here also highlight the importance of employing elements characterized by larger electronegativity, smaller atomic radius, and a lower number of valence electrons in the p and d orbitals in designing superhard compounds to enhance the likelihood of achieving high hardness in the designed materials. Further details and insights obtained by using SHAP analysis are provided in the SI.

## 4 Conclusions

In summary, we demonstrate a machine-learning approach to accelerate the discovery of new superhard compounds comprising boron, carbon, and oxygen. Random forest models for the prediction of two proxy properties, i.e bulk and shear moduli, were developed, and the predictions were used to estimate the hardness using Tian’s empirical formula. The ML models were applied further to screen promising superhard candidates from a large set of hypothetical BCO compositions. More than 300 BCO compositions were identified to be potentially superhard, exhibiting a predicted hardness of more than 35 GPa. Applying the atomic-level structure search method, DFT computation, and stability-based filters, a handful of BCO compositions were selected. Four identified new and promising superhard compounds display superhardness and mechanical, thermodynamic, and dynamic stability. Additionally, key parameters influencing hardness have been identified. The approach adopted in this work can be extended easily to expand the chemical search space to discover other superhard compounds.

## Supporting Information Available

Optimized parameters of developed machine learning models for bulk (*K*) and shear (*G*) modulus; down-selection of B–C–O compositions for DFT validation; stability assessment for the compositions; structure details for B_4_C_7_O_1_ composition; summary of the structures corresponding to the identified superhard compositions; features analysis using SHapley Additive exPlanations (SHAP) (PDF)
