# Supplementary material for: Informatics-Driven Design of Superhard B–C–O Compounds
Source: ACS Appl Mater Interfaces. 2024 Feb 17;16(8):10372–9. doi: 10.1021/acsami.3c18105 (PMC10910474; doi:10.1021/acsami.3c18105)
Supplement: Supplementary file 1 — am3c18105_si_001.pdf [file am3c18105_si_001.pdf]

Supporting Information

Informatics-Driven Design of Superhard B-C-O Compounds

Madhubanti Mukherjee,[†] Harikrishna Sahu,[†] Mark D. Losego,[†] Will R. Gutekunst,[‡] and Rampi Ramprasad[*,†]

†*School of Materials Science and Engineering, Georgia Institute of Technology, Atlanta, Georgia 30332, United States*

‡*School of Chemistry and Biochemistry, Georgia Institute of Technology, Atlanta, Georgia 30332, United States*

E-mail: rampi.ramprasad@mse.gatech.edu

1.1 Optimized parameters of developed machine learning models for bulk (K) and shear (G) modulus

The hyper-parameters for the best-selected models are shown in Table S1.

Table S1: Optimized hyperparameters

**Target property**	**max_depth**	**n_estimator**	**RF random state**
Bulk modulus (K)	30	160	10
Shear modulus (G)	40	160	25

1.2 Down-selection of B-C-O compositions for DFT validation

The structure selection criteria employed in this study are summarized as follows:

- Initial Downselection: From a pool of 335 compositions predicted to have a hardness greater than 35 GPa, an initial downselection was made based on several criteria. These included the total number of valence electrons and the total number of atoms in each composition. Further refinement in the selection process involved restricting the number of atoms in the structures. This step was crucial in narrowing down the potential candidates.
- Final Selection Based on Symmetry and Optimization: Among the 19 structures that passed the initial filters, a detailed analysis based on symmetry and optimization was conducted. This analysis led to the identification of only four structures that met all the required criteria.

As explained in Figure S1, initially 19 B-C-O compositions were identified and downselected to 15 compositions for further DFT calculations. These are listed in Table S2.

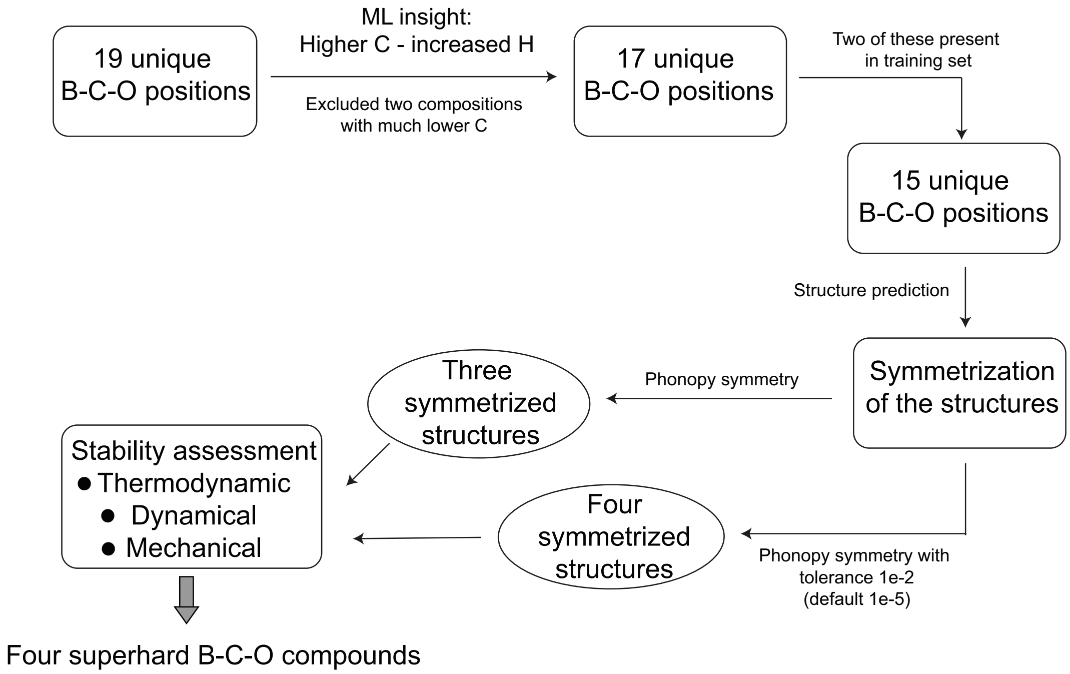

Figure S1: 15 superhard B-C-O compositions identified for further DFT validation by considering ML-informed decision, prior knowledge of compositions, symmetry search, leading to final four identified superhard B-C-O compounds after performing stability analysis.

Table S2: 15 unique B-C-O compositions

**Compositions**	**Symmetry search**
$B_4C_9O_3$	Not successful
$B_4C_8O_4$	Successful
$B_2C_{10}O_4$	Not successful
$B_6C_5O_5$	Not successful
$B_6C_6O_4$	Not successful
$B_4C_7O_5$	Not successful
$B_2C_9O_5$	Not successful
$B_6C_7O_3$	Not successful
$B_6C_8O_2$	Not successful
$B_2C_9O_1$	Successful
$B_2C_8O_2$	Successful
$B_2C_7O_3$	Successful
$B_4C_5O_3$	Successful
$B_2C_6O_4$	Successful
$B_4C_7O_1$	Successful

This study employed a multi-step selection process involving both quantitative criteria (like

valence electrons and atom count) and qualitative analysis (such as symmetry and optimization studies) to narrow down the vast pool of candidates to the most promising few. Among the seven symmetrized BCO structures, only five ($B_4C_8O_4$, $B_2C_9O_1$, $B_2C_8O_2$, $B_4C_5O_3$, and $B_4C_7O_1$) were optimized successfully. All the structures are dynamically stable, except $B_4C_5O_3$, leading to a final set of four BCO structures with predicted hardness (H_{pred}) more than 35 GPa. Among these four BCO compounds, $B_4C_7O_1$ has a DFT calculated hardness of 30.9 GPa, which is relatively lower than the other three compounds having calculated hardness of more than 40 GPa, thus identified as superhard.

1.3 Stability assessment for the compositions

For all the potential compositions the thermodynamic, mechanical, and dynamical stability have been verified. The thermodynamic stability has been determined by calculating the formation energy ΔE as shown below:

$$\Delta E = \frac{E(B_xC_yO_z) - xE(B) - yE(C) - zE(O)}{x + y + z} \tag{S1}$$

which is the difference between the total energy E of $B_xC_yO_z$ and the total energies of atomic C, B, and O.

The mechanical stability has been determined by using Born's criteria for the stability of monoclinic systems,[1,2] as explained:

$$(i)C_{11} > 0; (ii)C_{22} > 0; (iii)C_{33} > 0; (iv)C_{44} > 0; (v)C_{55} > 0; (vi)C_{66} > 0;$$

$$(vii)[C_{11} + C_{22} + C_{33} + 2 \times (C_{12} + C_{13} + C_{23})] > 0; (viii)C_{33} \times C_{55} - (C_{35})^2 > 0;$$

$$(ix)C_{44} \times C_{66} - (C_{46})^2 > 0; (x)C_{22} + C_{33} - 2 \times (C_{23}) > 0$$

$$(xi)C_{22} \times (C_{33} \times C_{55} - (C_{35})^2) + 2 \times C_{23} \times C_{25} \times C_{35} - ((C_{23})^2) \times C_{55} - ((C_{25})^2) \times C_{33} > 0$$

$$(xii) 2 \times [C_{15} \times C_{25} \times (C_{33} \times C_{12} - C_{13} \times C_{23}) + C_{15} \times C_{35} \times (C_{22} \times C_{13} - C_{12} \times C_{23})$$

$$+ C_{25} \times C_{35} \times (C_{11} \times C_{23} - C_{12} \times C_{13})] - [C_{15} \times C_{15} \times (C_{22} \times C_{33} - (C_{23})^2) + C_{25} \times C_{25} \times (C_{11} \times C_{33} - (C_{13})^2)$$

$$+ C_{35} \times C_{35} \times (C_{11} \times C_{22} - (C_{12})^2)] + C_{55} \times g > 0,$$

$$where, g = [C_{11} \times C_{22} \times C_{33} - C_{11} \times C_{23} \times C_{23} - C_{22} \times C_{13} \times C_{13} - C_{33} \times C_{12} \times C_{12} + 2 \times C_{12} \times C_{13} \times C_{23}]$$

The formation energy and the Born stability criteria have been calculated for all of the identified BCO compositions in a similar manner. The dynamical stability has been assessed by calculating the phonon spectra for the identified compositions.

1.4 Structure details for $B_4C_7O_1$ composition

$B_4C_7O_1$ crystallizes in a monoclinic structure, for which the thermodynamic, mechanical, and dynamical stability have been verified. This system is mechanically and dynamically stable with

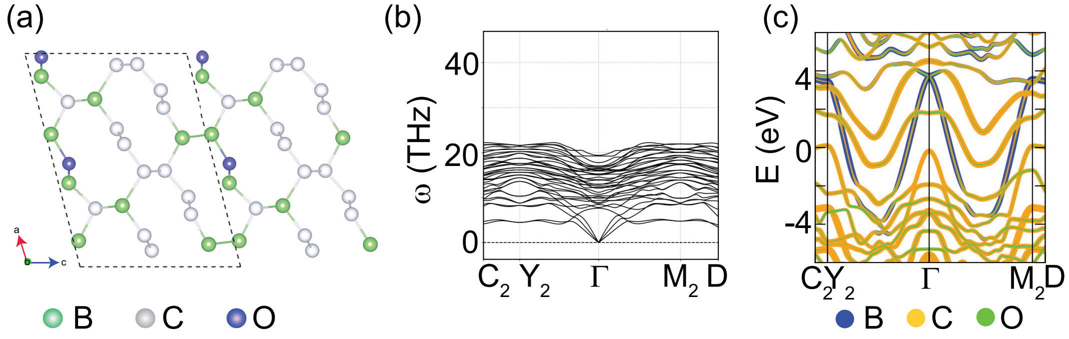

Figure S2: Monoclinic structure of (a) $B_4C_7O_1$ and corresponding (b) phonon dispersion, and (c) electronic band structure

a formation energy of 0.014 eV/atom. However, $B_4C_7O_1$ does not exhibit bandgap within the considered framework of calculating electronic band structure in this study. The DFT calculated and ML predicted bulk (K) and shear (G) modulus and estimated hardness are shown in Table S3.

1.5 Summary of the structures corresponding to the identified superhard compositions

This study identifies four superhard B-C-O compounds, which include $B_4C_8O_4$, $B_2C_9O_1$, $B_2C_8O_2$, $B_1C_{10}O_1$. These systems have been tested for thermodynamic, mechanical, and dynamical stability by calculating the formation energy, using Born criteria, and phonon dispersions through DFT. The summary is shown in Table S3.

Table S3: ML predicted and DFT calculated properties

**Name**	$\mathbf{K}_{ML}$ **(GPa)**	$\mathbf{K}_{DFT}$ **(GPa)**	$\mathbf{G}_{ML}$ **(GPa)**	$\mathbf{G}_{DFT}$ **(GPa)**	$\mathbf{H}_{ML}$ **(GPa)**	$\mathbf{H}_{DFT}$ **(GPa)**	ΔE_{calc} **(eV/atom)**
$B_4C_8O_4$	308.9	304.2	268.8	282.8	41.1	45.9	-0.049
$B_2C_9O_1$	334.7	332.3	343.6	336.6	59.2	57.4	0.10
$B_2C_8O_2$	316.7	259.7	285.3	239.0	44.7	40.4	0.11
$B_1C_{10}O_1$	348.2	366.9	330.6	360.4	52.7	55.5	1.98
$B_4C_7O_1$	308.4	220.7	249.7	187.0	36.0	30.9	0.014

1.6 Features analysis using SHapley Additive exPlanations (SHAP)

Interpreting the descriptors employed in the ML models facilitates the derivation of design guidelines, offering useful insights and rational pathways to accelerate the discovery of new promising materials that meet the desired target requirements. Hence, to analyze the local and global effects of the features on the predicted target values, SHAP analysis was performed. The findings can be summarized as follows:

- The feature importance for bulk and shear modulus as shown in Figures S3 (a) and (b), respectively, represent minimum electronegativity (X_{min}), fraction weighted mean of atomic radius (r_{fwm}), and fraction weighted mean of the number of valence electrons in p orbital (p^{val}_{fwm}) as the most important features for the target properties.

- Figure S4 (a) and (b) show bulk and shear modulus to have a direct dependence on X_{min}.

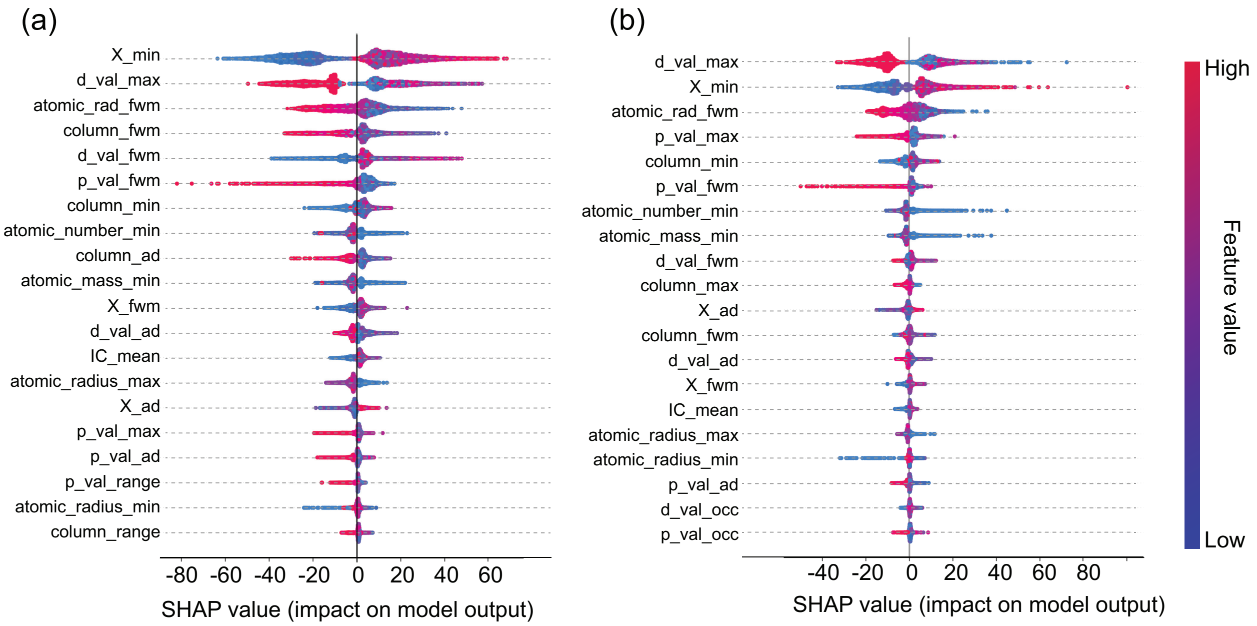

Figure S3: Global interpretability of the features SHAP, where (a) SHAP feature importance plot for bulk modulus (K) and (b) SHAP feature importance plot for shear modulus (G), respectively.

- Figures S5 (a) and (b) demonstrate the role of atomic radius in determining bulk and shear modulus. A smaller crystal unit cell favors a higher bulk and shear modulus due to a tightly packed system, thereby increasing hardness.

- Further analysis shows that increasing the number of valence electrons in the p orbital could affect the elastic moduli, in turn, the hardness negatively, as shown in Figures S6 (a) and (b). This confirms the direct and inverse influence of X_{min} and p_{fwm}^{val}, respectively. In addition, this agrees well with the fact that increasing oxygen contents in BCO compositions leads to smaller hardness.

- Local interpretability was also analyzed through individual SHAP plots, validating the impact of specific features such as X_{min} on the model output, as shown in Figure S7 (a) and (b). Two samples from the training set can be considered, namely $BeSiIr_2$ and $Li_2Ge_4N_6$ with bulk modulus 344 GPa and 125 GPa, respectively. For $BeSiIr_2$, X_{min} pushes the predicted value of bulk modulus towards the higher side of the base value (mean of the target value over the train data), as shown in Figure S8 (a), leading to a higher K, validating the positive

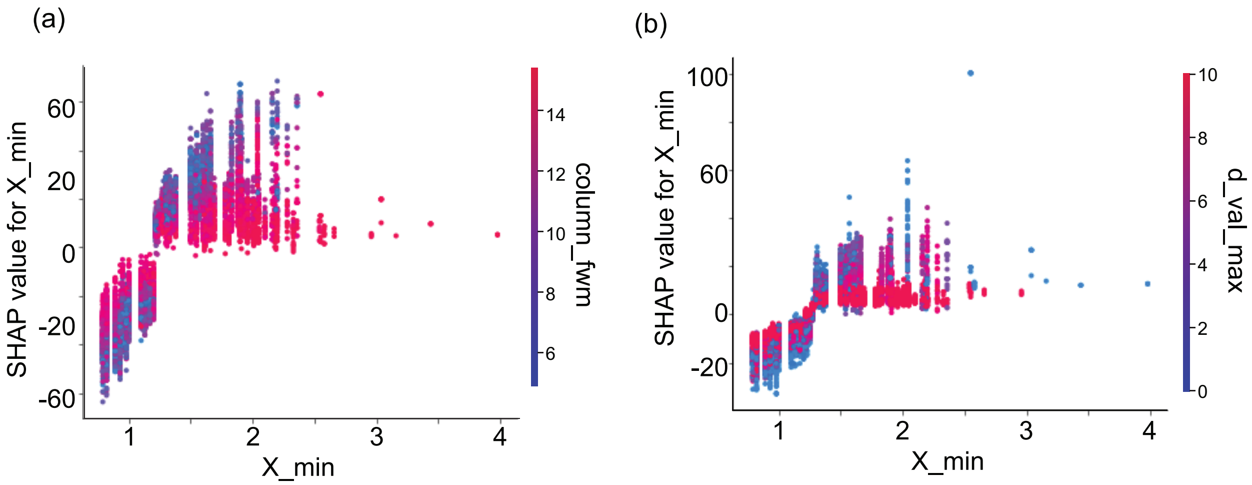

Figure S4: SHAP dependence plot for minimum electronegativity (X_min) for (a) bulk (K) and (b) shear (G) modulus, respectively.

correlation between X_{min} and K. On the other hand, Figure S8 (b) shows the value of X_{min} for $Li_2Ge_4N_6$ is low and pushes the predicted K towards the lower side. Thus, individual features impact the determination of the model outputs.

In short, to design superhard compounds, we strongly recommend utilizing elements with larger electronegativity, smaller atomic radius, and lower number of valence electrons in p and d orbitals.

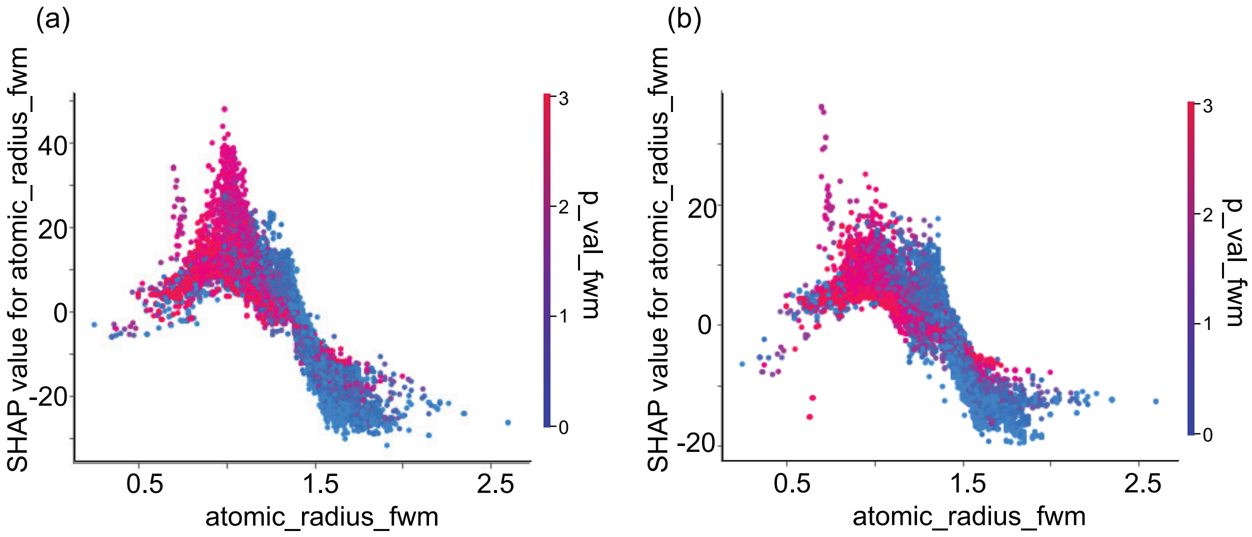

Figure S5: SHAP dependence plot for fraction weighted mean of atomic radius (atomic_radius_fwm) for (a) bulk (K) and (b) shear (G) modulus, respectively.

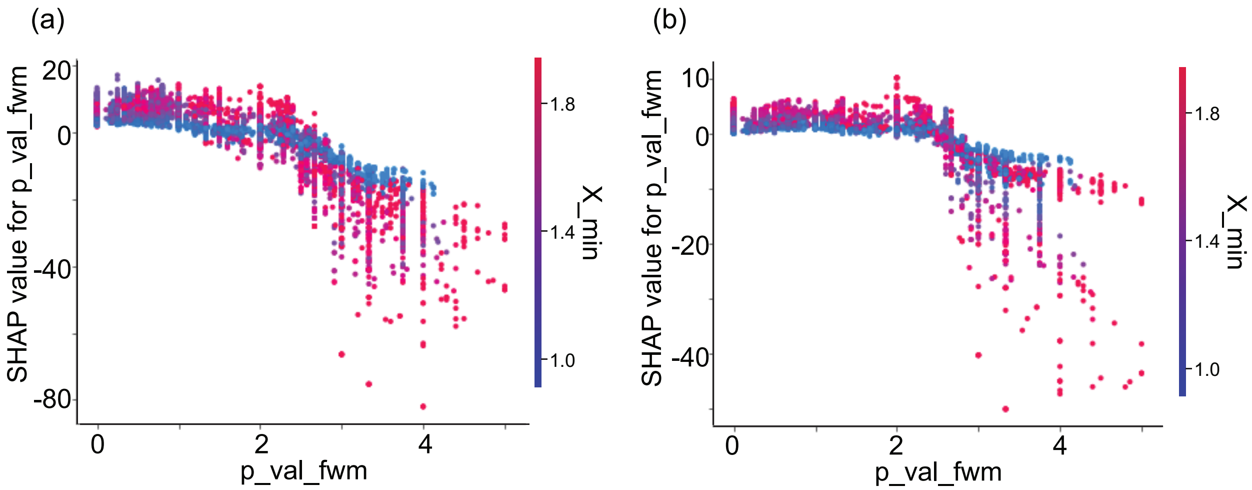

Figure S6: SHAP dependence plot for fraction weighted mean of the number of valence electrons in p orbital (p_val_fwm) for (a) bulk (K) and (b) shear (G) modulus, respectively.

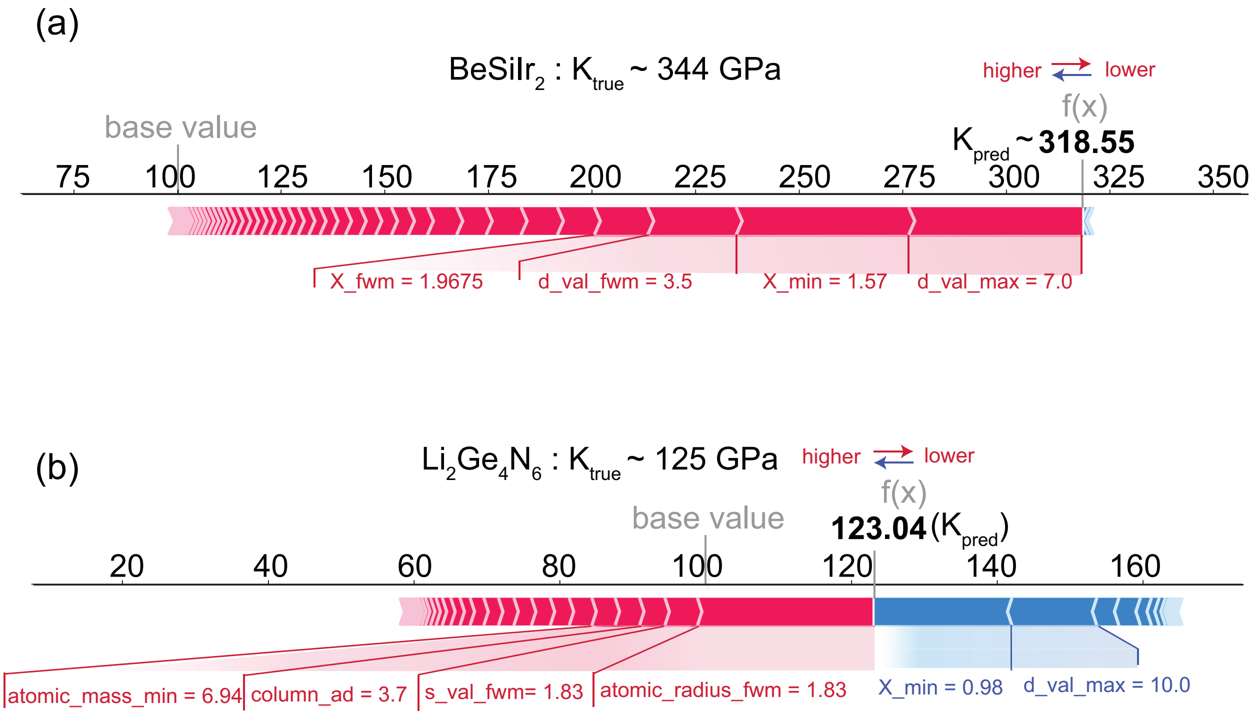

Figure S7: Local interpretability into bulk modulus using SHAP: individual SHAP plots for (a) $BeSiIr_2$ and (b) $Li_2Ge_4N_6$. The numbers below the features denote their values for a particular observation, predicted values are shown under f(x), and the base value (100 GPa) is the mean of the model output over the train data. The features that push the predicted value higher (to the right) are shown in red, and those pushing the prediction lower (to the right) are shown in blue. For a particular observation, if the value of a feature that has a positive (negative) impact on model output is more than its mean value, then it will push the base value towards the right (left).

References

(1) Mouhat, F.; Coudert, F.-X. Necessary and sufficient elastic stability conditions in various crystal systems. *Phys. Rev. B* **2014**, *90*, 224104.

(2) Wu, Z.-j.; Zhao, E.-j.; Xiang, H.-p.; Hao, X.-f.; Liu, X.-j.; Meng, J. Crystal structures and elastic properties of superhard Ir N 2 and Ir N 3 from first principles. *Phys. Rev. B* **2007**, *76*, 054115.
